# Hyperpolarized Carbon-13 MRI in Breast Cancer

**DOI:** 10.3390/diagnostics13132311

**Published:** 2023-07-07

**Authors:** Ramona Woitek, Kevin M. Brindle

**Affiliations:** 1 Research Centre for Medical Image Analysis and AI, Danube Private University, 3500 Krems, Austria; 2 Department of Radiology, University of Cambridge, Cambridge CB2 0QQ, UK; 3 Cancer Research UK Cambridge Centre, University of Cambridge, Cambridge CB2 0RE, UK; kevin.brindle@cruk.cam.ac.uk; 4 Cancer Research UK Cambridge Institute, Li Ka Shing Centre, University of Cambridge, Cambridge CB2 0RE, UK; 5 Department of Biochemistry, University of Cambridge, Cambridge CB2 1QW, UK

**Keywords:** magnetic resonance imaging, hyperpolarization, carbon-13, metabolism, breast cancer

## Abstract

One of the hallmarks of cancer is metabolic reprogramming, including high levels of aerobic glycolysis (the Warburg effect). Pyruvate is a product of glucose metabolism, and ^13^C-MR imaging of the metabolism of hyperpolarized (HP) [1-^13^C]pyruvate (HP ^13^C-MRI) has been shown to be a potentially versatile tool for the clinical evaluation of tumor metabolism. Hyperpolarization of the ^13^C nuclear spin can increase the sensitivity of detection by 4–5 orders of magnitude. Therefore, following intravenous injection, the location of hyperpolarized ^13^C-labeled pyruvate in the body and its subsequent metabolism can be tracked using ^13^C-MRI. Hyperpolarized [^13^C]urea and [1,4-^13^C_2_]fumarate are also likely to translate to the clinic in the near future as tools for imaging tissue perfusion and post-treatment tumor cell death, respectively. For clinical breast imaging, HP ^13^C-MRI can be combined with ^1^H-MRI to address the need for detailed anatomical imaging combined with improved functional tumor phenotyping and very early identification of patients not responding to standard and novel neoadjuvant treatments. If the technical complexity of the hyperpolarization process and the relatively high associated costs can be reduced, then hyperpolarized ^13^C-MRI has the potential to become more widely available for large-scale clinical trials.

## 1. Background

One of the hallmarks of cancer is metabolic reprogramming [1,2]. Tumors frequently exhibit high levels of aerobic glycolysis (the Warburg effect), where increased glycolytic flux can provide the biosynthetic intermediates required for cell proliferation [3]. A widely used clinical imaging technique that can be used to detect this phenomenon is 2-deoxy-2-[^18^F]fluoroglucose (FDG)-positron emission tomography (PET; [^18^F]FDG-PET). Following intravenous injection of the radioactive glucose analog, [^18^F]FDG, lesions with increased glucose uptake can be visualized with higher sensitivity and specificity than when using computed tomography (CT) [4,5]. These imaging modalities, which both involve ionizing radiation, can be combined for improved anatomic mapping of metabolically active lesions. In contrast, magnetic resonance imaging (MRI) offers higher soft tissue contrast than CT while being ionizing radiation-free. Especially in the diagnosis and local staging of breast cancer, it offers unique soft tissue contrast and high sensitivity and specificity for the detection and characterization of disease [6]. A metabolic imaging technique based on MRI that could be combined with conventional ^1^H MRI would address the need for a high degree of anatomical detail together with improved functional tumor phenotyping and accurate and timely response evaluation in breast cancer patients undergoing treatment. Magnetic resonance spectroscopy and spectroscopic imaging (MRS and MRSI) provide unique opportunities for metabolic imaging as they allow different metabolites to be distinguished based on the different resonance frequencies of their MR-active nuclei (such as ^1^H). However, with ^1^H MRS measurements, there is often no information about metabolic flux, and moreover, the metabolite resonances can overlap, making a detailed evaluation of single metabolites challenging. Lipid and lactate peaks in the breast are one example where intratumoral and peritumoral lipids make the evaluation of tumor lactate from ^1^H MR spectra extraordinarily difficult. In breast cancer and other tumors, phospholipid metabolism is frequently altered due to increased cell membrane turnover as a result of cell proliferation, which can lead to the detection of choline-containing compounds in ^1^H spectra [7,8,9]. Increased total choline (tCho) has been detected in malignant breast lesions but not in benign lesions or normal breast tissue, and decreases have been observed in response to neoadjuvant chemotherapy [10,11,12]. However, the use of ^1^H MRS for response assessment in breast cancer is limited by several factors, including limited sensitivity in the clinical setting, which requires lengthy scan times, and a requirement for data acquisition and analysis to be performed by specialists using methods that are not widely available [13]. In contrast to the spectral overlap observed with ^1^H MRS, ^13^C-MRS gives better-resolved spectra due to the generally larger chemical shift differences between metabolite ^13^C resonances. However, due to a low natural abundance of only 1.1% and a gyromagnetic ratio that is a quarter that of ^1^H, the spatial and temporal resolution of ^13^C-MRS or MRSI is very limited. Hyperpolarization of the ^13^C spin in a ^13^C-labeled molecule can increase the signal-to-noise ratio by 4–5 orders of magnitude. Therefore, following intravenous injection the location of the labeled molecule in the body and, more importantly, its subsequent metabolism can be tracked using ^13^C-MRS and ^13^C MRSI. Following a decade of preclinical development [14,15,16,17,18,19], this technique translated to the clinic in 2013 [20].

## 2. The Process of Hyperpolarization

**Figure 1:**
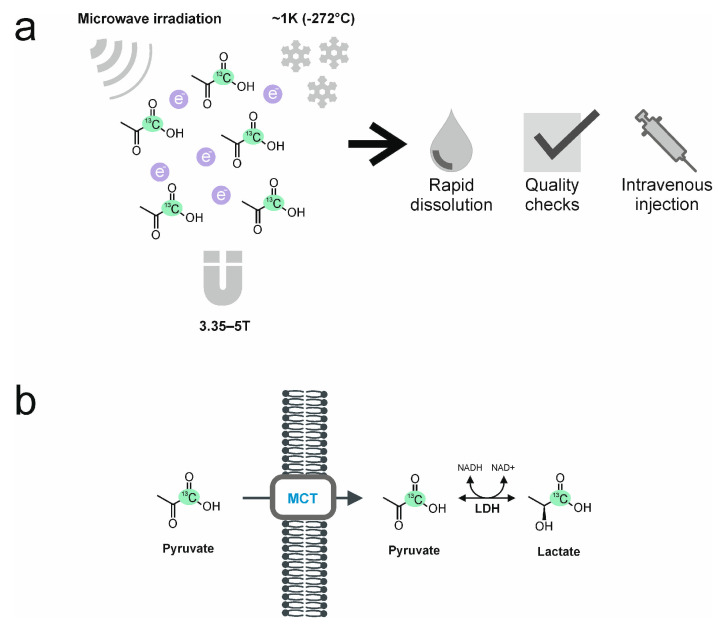
Dynamic Nuclear Polarization (DNP), and intracellular uptake and enzymatic conversion of [1-^13^C]pyruvate to lactate. (**a**) During DNP, the solution containing [1-^13^C]pyruvate, is doped with a stable radical containing unpaired electrons (electron paramagnetic agent, EPA), which become polarized when frozen at ~1 K (−272 °C) and exposed to a strong magnetic field (3.35–5 T). Microwave irradiation transfers spin polarization from the electron spins to the ^13^C nuclei. The hyperpolarized frozen sample is then rapidly dissolved using superheated water. Before intravenous injection into a cubital vein, the EPA is removed, and its concentration, as well as the pyruvate concentration, pH, and temperature, are assessed. (**b**) After intravenous injection and transit to the tissue of interest, the hyperpolarized [1-^13^C]pyruvate is taken up intracellularly by the monocarboxylate transporters (MCTs). Lactate dehydrogenase (LDH) in the cytosol catalyzes the exchange of the hyperpolarized ^13^C-label between pyruvate and the endogenous lactate pool.

The most widely used hyperpolarization technique, and the only technique that has been used thus far in the clinic, is dynamic nuclear polarization (DNP; Figure 1). In this technique, a solution containing an endogenous ^13^C-labeled metabolite, such as ^13^C-labeled pyruvate, is doped with a stable free radical (electron paramagnetic agent, EPA). The unpaired electrons in the EPA become polarized when the mixture is frozen at ~1 K (−272 °C) and exposed to a strong magnetic field (3.35–5 T). Microwave irradiation is then used to transfer spin polarization from the electron spins to the ^13^C spins. After this process, which can take more than an hour, the frozen sample is rapidly dissolved and brought to the liquid state using superheated water. The EPA is then removed, and its remaining concentration, as well as the concentration of the ^13^C-labeled metabolite in the hyperpolarized solution, together with pH and temperature, are assessed. After having passed these quality checks, the solution can be safely injected (intravenously) into the patient. A clinical hyperpolarizer typically reaches polarization levels of 20–30% for [1-^13^C] pyruvate in the liquid state, which is equivalent to an increase in signal of more than 10^4^-fold compared to the signal that would be obtained at thermal polarization at 3T [21,22,23,24]. A fundamental limitation of the technique is the short lifetime of the hyperpolarization, which in the case of [1-^13^C]pyruvate has a half-life in vivo of 20–30 s; therefore, the intravenous injection needs to be performed as quickly as possible, and images are typically acquired over the following 1–2 min with a temporal resolution of around 4 s.

The relatively high upfront cost of a clinical hyperpolarizer (in excess of $1 M) and the requirement to have an on-site pharmacy, and associated personnel, to load the device with the ^13^C-labeled probe has driven the development of alternative approaches to delivering hyperpolarized ^13^C-labeled probes in the clinic. These potentially could reduce costs and make the technique more widely available. One approach would be to centralize production of the hyperpolarized ^13^C-labeled substrate using the DNP technique and then ship the hyperpolarized material to the clinic at low temperature and moderately high magnetic field, conditions in which the hyperpolarization has a very long lifetime (of the order of hours). This has the additional benefits of potentially ensuring better quality control and uniformity of hyperpolarization. All the radiology department needs then is a device to rapidly thaw the frozen sample and bring it to room temperature for injection. Storage and subsequent transport of a hyperpolarized ^13^C-labeled probe can be achieved by replacing the EPA with UV-generated radicals, which can be thermally annihilated following the DNP process by transiently raising the temperature without significant loss of nuclear spin polarization. Removal of the radical is essential for the storage and transport of the polarized material since the continued presence of the radical will result in accelerated loss of polarization [25]. An alternative approach that does not require the very low temperatures needed for DNP and that can polarize the ^13^C-labeled probe much more rapidly and use a much more compact device is parahydrogen-induced hyperpolarization (PHIP) [26]. In this technique, parahydrogen is reacted with a precursor molecule, and the spin order in the parahydrogen is transferred to the spin-coupled ^13^C nucleus in a process that can take place at room temperature. Initially, very few molecules that would be of interest for metabolic studies could be polarized using the technique. However, this restriction was lifted by Aime and co-workers who showed with pyruvate, for example, that the carboxyl carbon could be hyperpolarized by PHIP by using a precursor containing a hydrogenable functionality (“side arm”) that could be cleaved off following the transfer of polarization to regenerate free pyruvate [27]. A commercial preclinical PHIP device that uses side arm technology is currently available, with a clinical device planned for the near future (https://www.nvision-imaging.com/; accessed on 20 June 2023). An alternative approach to metabolic imaging that is easier to implement since it dispenses with the need for a hyperpolarization device is to use ^2^H-labeled substrates. Despite early work with ^2^H-labeled substrates in the 1980s, the nucleus attracted little subsequent interest for metabolic imaging due to its relatively low sensitivity of detection and narrow spectral frequency range, which can result in poorly resolved spectra, a problem compounded by the nucleus’s quadrupolar moment which can lead to broad resonances. However, the quadrupolar moment, which can result in very short T_1_ relaxation times, means that the low sensitivity of detection can be compensated by rapid signal acquisition in the absence of signal saturation. The publication of a landmark paper in 2018 [28], in which it was shown that oral administration of [6,6′-^2^H_2_]glucose resulted in detectable 2H labeling of lactate (a measure of glycolytic flux) and glutamate/glutamine (Glx) (a surrogate for flux in the mitochondrial TCA cycle) in the brains of glioblastoma patients stimulated renewed interest in this nucleus (reviewed in [29,30]. The tumor showed higher lactate labeling and lower Glx labeling than the surrounding normal-appearing brain tissue. Clinical image resolutions are comparable with those obtained with hyperpolarized ^13^C-labeled metabolites have been achieved, albeit at relatively high magnetic fields. In addition to ease of use, since deuterated substrates can simply be taken off the shelf, they have the additional advantage over ^13^C-labeled substrates that they tend to be less expensive to produce, although this advantage is offset to some extent by the larger amounts that need to be administered [30].

## 3. Hyperpolarized [1-^13^C]pyruvate

The most frequently used ^13^C-labeled molecule in preclinical studies and the only substrate that has been used thus far in clinical studies is [1-^13^C]pyruvate, which is the focus of this review. As a breakdown product of glucose and at the end of the glycolytic pathway, it occupies a key position in cellular metabolism: it can either enter the mitochondria and the tricarboxylic acid cycle, where it undergoes oxidation or be reduced to lactate in the reaction catalyzed by the enzyme lactate dehydrogenase (LDH) in the cytosol. The ^13^C-label is most frequently introduced in the carboxyl position since, in this position, it has a long T_1_ at clinical magnetic field strengths, which means that the hyperpolarization is relatively long-lived, lasting between one to two minutes in vivo [20]. Following intravenous injection into a cubital vein and transit to the tissue of interest, the hyperpolarized [1-^13^C]pyruvate is taken up into cells by the monocarboxylate transporters (MCTs) where LDH catalyzes the exchange of the hyperpolarized ^13^C-label between pyruvate and the endogenous lactate pool (Figure 1b).

## 4. In Vitro and In Vivo Experiments with HP ^13^C-MRI in Breast Cancer

One of the main motivations for the clinical translation of HP ^13^C-MRI is the expectation that this will provide a more accurate method for detecting early treatment response than currently used clinical imaging techniques. Numerous preclinical studies have demonstrated the potential of HP ^13^C-MRI for detecting early treatment response (Table 1). For example, ^13^C-MRI with hyperpolarized [1-^13^C]pyruvate and [^18^F]FDG-PET were compared directly for assessing treatment response in human colorectal (Colo205) and breast adenocarcinoma (MDA-MB-231) xenografts in mice treated with an apoptosis-inducing agent (TRAIL agonist) [31]. At only 24 h after treatment, there was a decrease in lactate labeling, whereas [^18^F]FDG uptake remained unchanged. Increasing tumor macrophage infiltration during treatment has been suggested as a potential contributor to stable or increasing [^18^F]FDG uptake. However, in this example, this could not account fully for the lack of change in [^18^F]FDG uptake due to the sparsity of tumor-infiltrating immune cells. 

**Figure 2:**
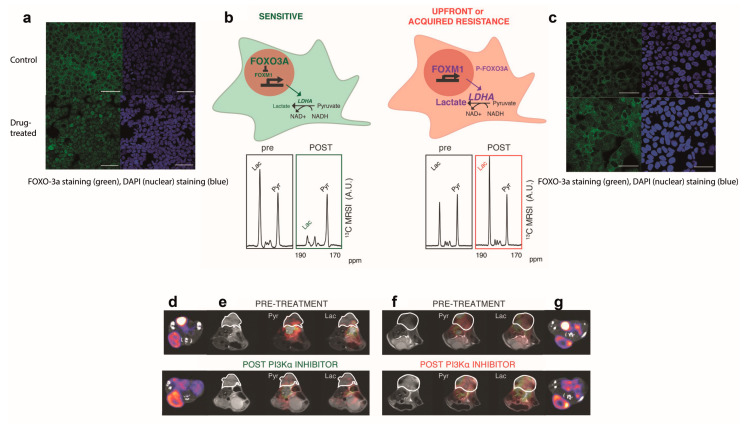
Imaging with hyperpolarized [1-^13^C]pyruvate detects response and resistance to treatment with a PI3K inhibitor in ER+ breast cancer patient-derived xenografts. In drug-sensitive xenografts, the PI3K inhibitor inhibited phosphorylation of FOXO3A, which migrates into the nucleus inhibiting the expression of the transcription factor FOXM1, which in this tumor type drives the expression of LDHA. The resulting decrease in LDHA expression results in decreased hyperpolarized ^13^C label exchange between injected [1-^13^C]pyruvate and the endogenous tumor lactate pool. There was no change in the expression of the c-Myc and HIF-1α transcription factors, which can explain why there was no change in [^18^F]FDG uptake. In drug-resistant tumors, phosphorylation of FOXO3A led to its retention in the cytosol and sustained expression of FOXM1 and LDHA, and sustained labeling of lactate from hyperpolarized [1-^13^C]pyruvate. (**a**,**c**) fluorescence microscopy images showing migration of FOXO3A into the nucleus in a drug-sensitive tumor. (**b**) scheme showing control of LDHA expression by FOXO3A and localized ^13^C spectra showing a decrease in lactate labeling in the drug-sensitive tumor posttreatment. The lactate and pyruvate peaks are labeled. Spectra were acquired at 7.0-T using a ^13^C/^1^H volume transmit coil with a 20 mm diameter ^13^C receiver coil. (**d**,**g**) [^18^F]FDG-PET images overlaid on CT images in drug-sensitive and drug-resistant tumors before and after treatment. The tumors are in the bottom left quadrant, and the images show no change in [^18^F]FDG uptake post-treatment. (**e**,**f**) Hyperpolarized ^13^C-labeled metabolite false-color images overlaid on T_2_-weighted ^1^H images. The tumor is outlined. ^13^C images were acquired using a 3D single-shot sequence that used spectral-spatial pulses for selective excitation of the pyruvate and lactate resonances. Figure adapted from figures in [36].

HP ^13^C-MRI has also been shown to outperform [^18^F]FDG-PET in detecting resistance to targeted breast cancer treatment. In patient-derived ER+ breast cancer xenografts (PDXs) treated with a PI3Kα inhibitor ^13^C-label exchange between pyruvate and lactate was decreased in drug-sensitive but not in drug-resistant tumors, whereas [^18^F]FDG uptake was unaffected in both the drug-sensitive and resistant tumors [36]. The sustained ^13^C-label exchange between pyruvate and lactate in the drug-resistant tumors was explained by the persistent expression of the transcription factor FOXM1, which in these ER+ breast cancer PDXs was shown to drive the expression of LDHA (Figure 2). These findings suggest that imaging with hyperpolarized [1-^13^C]pyruvate could be used as a companion diagnostic for the early assessment of treatment response and resistance in ER+ breast cancer patients undergoing targeted treatment with PI3Kα inhibitors. 

Multiple factors can affect the rate of ^13^C-label exchange between the injected pyruvate and the endogenous lactate pool, including delivery of pyruvate to the tissue, expression of LDH and the monocarboxylate transporters (MCTs), and the intracellular concentrations of pyruvate and lactate and the coenzymes NADH and NAD^+^, which will determine the activity of LDH in the cell [15]. For example, a control analysis in EL4 murine lymphoma cells showed that the MCTs and LDH have nearly equal flux control coefficients for the exchange [32]. In contrast, a mass spectrometry imaging study on EL4 tumor sections showed that there was no correlation between the distribution of ^13^C-labeled and unlabeled lactate in the tumors. However, there was a strong correlation between labeled pyruvate and lactate in the corresponding ^13^C MR images acquired in vivo, suggesting that in the EL4 tumor, pyruvate delivery is more important [37]. The relative importance of pyruvate delivery and transport and lactate dehydrogenase activity in determining the rate of hyperpolarized ^13^C label exchange between pyruvate and lactate appears to vary between different tumor types. A corollary of this is that the sensitivity of the exchange to inhibition of these different steps will also vary between tumor types. In the case of breast cancer, studies on TD47D (Luminal A) human breast cancer cells showed that pyruvate transport via MCT1 was rate-limiting for hyperpolarized ^13^C label exchange between pyruvate and lactate and that MCT1 inhibition reduced lactate labeling [38], whereas in two murine breast cancer models, lactate labeling was correlated with total LDH activity in the tumor but not MCT1 expression [39]. In experiments on cells from tumors with differing malignancies, triple-negative breast cancer cells (MDA-MB-231) showed lower lactate labeling than hormone receptor-positive cells (MCF-7 cells) [40], although this is contrary to what has been observed in patients, where triple-negative tumors showed greater lactate labeling than ER+ disease [33]. Lactate labeling in these cells was shown not to depend on MCT or LDH expression but on the glucose and glutamine concentrations in the cell media. In a comparison of highly metastatic (4T1) and metastatically dormant murine breast cancer models (4T07), lactate labeling was slightly (but not significantly) higher in the highly metastatic model [39].

## 5. Outlook for New Substrates for Clinical Translation

^13^C-labeled urea and fumarate have attracted interest as agents for imaging tissue perfusion and post-treatment necrotic cell death, respectively.

HP ^13^C urea is metabolically inactive and remains largely in the extracellular space. Therefore, HP ^13^C-urea maps reflect blood flow, tissue perfusion, and the volume of distribution [41,42]. Preclinical dynamic scanning after the intravenous injection of co-polarized [1-^13^C]pyruvate and [^13^C]urea or [^13^C, ^15^N_2_]urea have demonstrated the feasibility of acquiring both metabolic and perfusion maps simultaneously [43]. In TRAMP (Transgenic Adenocarcinoma of Mouse Prostate) mice [41], scanning after 1, 4, and 7 days of radiotherapy revealed a decrease in lactate-to-pyruvate conversion with a concomitant increase in the hyperpolarized urea signal [41]. The results obtained with hyperpolarized [^13^C]urea were similar to those obtained using dynamic contrast-enhanced (DCE) ^1^H MRI. One advantage of ^13^C-urea MRI is that it allows the normalization of the ^13^C-lactate and ^13^C-pyruvate signals. Therefore, it can improve a quantitative metabolic analysis as the results are no longer affected by tissue perfusion and pyruvate delivery. The clinical translation of ^13^C-MRI using co-polarized [1-^13^C]pyruvate and [^13^C, ^15^N_2_]urea is currently being pursued [43].

Fumarate is a TCA cycle intermediate and is converted to malate in a hydration reaction catalyzed by the enzyme fumarase, which exists as both cytosolic and mitochondrial isoforms. In necrotic cells, loss of plasma membrane integrity leads to the increased access of injected hyperpolarized [1,4-^13^C_2_]fumarate to fumarase and subsequently increased production of ^13^C-labeled malate [44]. Initial studies in lymphoma cells and in lymphoma-bearing mice showed that treatment-induced tumor cell necrosis resulted in a large increase in ^13^C-labeled malate production [44]. The potential for early response assessment in breast cancer was demonstrated by injecting co-polarized ^13^C-labeled pyruvate and fumarate into suspensions of MDA-MB-231 breast cancer cells and into mice with subcutaneous MDA-MB-231 tumors following their treatment with doxorubicin [15]. Treatment-induced cell death was accompanied by a decrease in the ^13^C-label exchange between [1-^13^C]pyruvate and lactate and a concomitant increase in flux between fumarate and malate, which in the tumors occurred before there were changes in the tumor size. A similar study was subsequently conducted in an untreated breast cancer cell line (MCF-7) [45], where fumarate-to-malate conversion was only detectable in necrotic cells. Recent studies have shown that necrotic cell death can also be detected using ^2^H-MRSI measurements of the conversion of [2,3-^2^H_2_]fumarate to [2,3-^2^H_2_]malate [46]. At only 48 h after treatment with a TRAIL agonist, the malate-to-fumarate ratio increased significantly in human breast cancer (MDA-MB-231) xenografts. This increase was correlated with increased levels of cell death. High magnetic field strengths (7T) have been used to compensate for the narrow frequency range of ^2^H-labeled metabolites [46]. However, clinical ^2^H-MRSI has been shown to be feasible at 3T. Following oral administration of [6,6′-^2^H_2_]glucose to healthy volunteers, maps of ^2^H-labeled water, glucose, lactate, and glutamate/glutamine (Glx) in the brain could be generated [47]. However, clinical feasibility in breast cancer has yet to be demonstrated.

## 6. Clinical Hyperpolarized ^13^C-MRI in Patients with Breast Cancer

Neoadjuvant chemotherapy (NACT) prior to surgery is the primary standard-of-care treatment option for up to 40% of patients with early-stage breast cancer, especially for patients with HER2+ and triple-negative breast cancer (TNBC) [48]. NACT can be used to downstage locally advanced breast cancer to enable breast conservation and allows direct assessment of response to well-established treatment regimens or to new drugs. Pathological complete response (pCR) at surgery is associated with a more favorable prognosis than non-pCR. However, a recent meta-analysis has shown that pCR rates in trials vary and range from 16.7% to 67.0% [49]. ^1^H-MRI, including DCE MRI, is used for the evaluation of pCR after completion of NACT and prior to surgery but has shown a limited pooled sensitivity of 0.67 [95% CI 0.58–0.74] and a slightly better-pooled specificity of 0.85 [95% CI 0.81–0.88] for HR−/HER2− breast cancer in a recent meta-analysis [49]. Earlier and more accurate detection of non-pCR in patients with breast cancer could guide decisions regarding a change in treatment in non-responders or could even allow treatment de-escalation if an early response could be identified reliably [34,35,50]. Based on the encouraging results of many preclinical studies, hyperpolarized ^13^C-MRI is thought to be one of the most promising candidate techniques for improving the accuracy of early response assessment for patients with breast cancer undergoing NACT. Together with evaluating the metabolic tumor phenotype noninvasively, this is the main driver behind the clinical translation of ^13^C-MRI for breast cancer.

**Figure 3:**
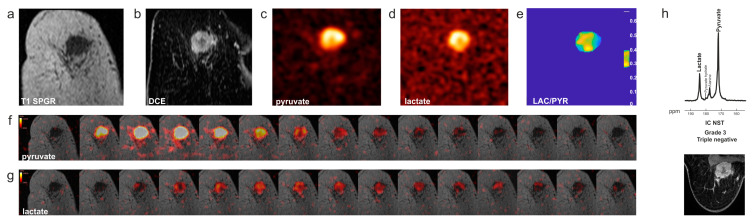
Triple-negative breast cancer on HP ^13^C-MRI. (**a**) Coronal T_1_ 3D spoiled gradient echo (SPGR) MR image. (**b**) Coronally reformatted DCE image at peak enhancement after intravenous injection of a gadolinium-based contrast agent. (**c**) Summed hyperpolarized ^13^C-pyruvate and (**d**) summed hyperpolarized ^13^C-lactate images. The area of low ^13^C-pyruvate and ^13^C-lactate signals in the center of the tumor is likely to correspond to an area with low enhancement on DCE MRI. (**e**) LAC/PYR map showing intratumoral heterogeneity. The dominant intratumoral heterogeneity was concordant between the DCE-MRI and hyperpolarized ^13^C-MR images and represents decreased delivery of both the gadolinium-based contrast agent and ^13^C-pyruvate to the center of the tumor. (**f**,**g**) Dynamic hyperpolarized ^13^C-pyruvate and ^13^C-lactate images acquired over 15 time points after intravenous injection of hyperpolarized [1-^13^C]pyruvate (delay = 12 s; temporal resolution = 4 s). (**h**) Top: ^13^C metabolite spectrum from a coronal dynamic IDEAL spiral CSI slice covering the tumor summed over 15 time points; Bottom: The axial image from the equivalent DCE-MRI data was taken at the time point of maximum tumor enhancement. Abbreviations: ppm parts per million; IC NST invasive cancer of no specific type. Figure reproduced with permission from [23,33].

The first clinical studies using hyperpolarized [1-^13^C]pyruvate in breast cancer were published in 2019 and 2020 and demonstrated feasibility in patients with early-stage disease [33,51] (Figure 3). Intertumoral metabolic heterogeneity was observed between different histological cancer subtypes (invasive lobular cancer and invasive cancer of no specific type), between molecular subtypes (hormone receptor-positive (HR+) and human epidermal growth receptor 2 negative (HER2-) and triple-negative breast cancers (TNBC; HR-/HER2-)) and between tumor grades [33]. The labeled lactate-to-pyruvate ratio was correlated with the expression of MCT1, LDHA, and HIF1 determined by immunohistochemistry and RNA sequencing of tumor biopsy samples [33]. Analysis of the METABRIC transcriptomic dataset, which includes clinical data and biopsy samples from around 2000 patients with breast cancer [52,53], showed that the expression of LDHA and MCT1 was significantly correlated with the expression of the hypoxia markers HIF1A and CAIX. Overexpression of LDHA and CAIX was also associated with poorer overall survival and progression-free survival. Therefore, the results obtained from hyperpolarized ^13^C-MRI may also give prognostic information [54]. This initial study showed that in breast cancers of higher nuclear grade and especially in TNBCs, ^13^C-labeled lactate is detectable at baseline, indicating that a potential decrease in the lactate-to-pyruvate ratio as a sign of treatment response should be detectable too. A first proof-of-principle study in a patient with TNBC undergoing platinum-based neoadjuvant chemotherapy showed that a decrease in the lactate-to-pyruvate ratio and in the apparent first-order rate constant describing label flux from pyruvate to lactate (*k*_PL_) could be observed as early as after one cycle of treatment [55]. While results from DCE-MRI and pharmacokinetic modeling indicated poor response (based on an increase in *K*^trans^), the early metabolic response seen with hyperpolarized ^13^C-MRI was eventually confirmed after the completion of seven cycles of NACT when pCR was identified from the surgical specimen. 

**Figure 4:**
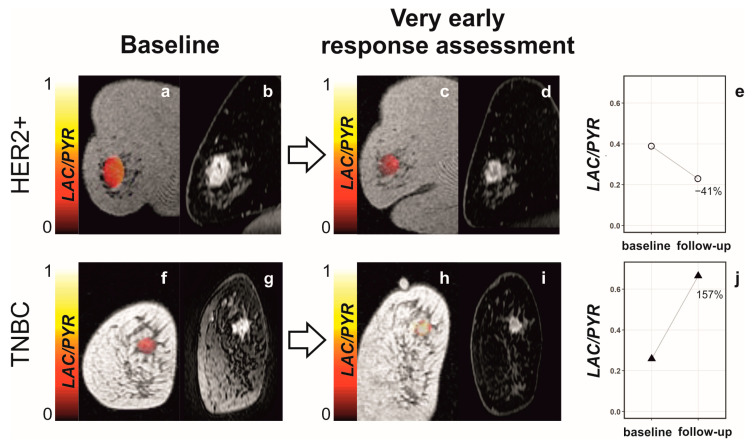
Changes in the lactate-to-pyruvate ratio between baseline and following treatment in responding and nonresponding breast cancer patients. (**a**,**c**,**f**,**h**) Coronal T_1_-weighted 3D spoiled gradient echo (SPGR) images with the lactate-to-pyruvate ratio map overlaid on the breast tumor. (**b**,**d**,**g**,**i**) Coronal reformatted DCE images were obtained 150 s after intravenous injection of a gadolinium-based contrast agent. A patient with HER2^+^ breast cancer was imaged at baseline (**a**,**b**) and for ultra-early response assessment (**c**,**d**) following standard-of-care treatment and showed a decrease in the lactate-to-pyruvate ratio of 41% (**e**). At surgery, non-pCR with residual invasive cancer was identified. Another patient with TNBC was imaged at baseline (**f**,**g**) and for ultra-early response assessment (**h**,**i**) following treatment with chemotherapy and a PARP inhibitor and showed an increase in the lactate-to-pyruvate ratio of 157% (**j**). At surgery, pCR without residual invasive breast cancer was found. Reprinted after modifications with permission from [54].

The response was detected at an even earlier time point in a subsequent study published in 2021, where a response was detected as early as 7–11 days into treatment with taxanes and the Poly (ADP-ribose) polymerase (PARP) inhibitor, Olaparib [54] (Figure 4). In a cohort of patients with TNBC and HER2+ breast cancers, changes in the lactate-to-pyruvate ratio predicted which patients would eventually reach pCR, while DCE MRI with pharmacokinetic modeling and DWI did not allow this distinction. Interestingly, it was an early increase in the lactate-to-pyruvate ratio of ≥20% that was common among patients with pCR (Figure 4j). This finding is contrary to the majority of studies in preclinical tumor models and measurements in cell culture, where a decrease in lactate labeling is usually observed post-treatment. However, a small proportion of studies have found an increasing lactate-to-pyruvate ratio as a sign of response in vivo and in vitro [56,57,58,59]. In preclinical studies with antiangiogenic drugs, an increase in the lactate-to-pyruvate ratio has been attributed to increased levels of hypoxia. Hypoxia can increase the expression of the plasma membrane transporter MCT1, which mediates cell uptake of pyruvate. MCT1 has been shown to be limiting for hyperpolarized [1-^13^C]lactate formation in breast cancer cell lines and in prostate cancer [38,57,60] and can also increase the concentrations of the coenzyme NADH and LDH, both of which have been shown to correlate with hyperpolarized lactate labeling [14,15,31,36,61]). Antiangiogenic effects have been observed with chemotherapeutic agents such as the taxanes used in this treatment response study. The PARP inhibitor, Olaparib, may also have contributed to increased lactate labeling by preserving the cellular NAD+/NADH pool following treatment. PARP1 and 2 sense DNA damage and facilitate DNA repair by using NAD+ as a substrate. PARP inhibitors impair protein PARylation (poly ADP-ribosylation), a process that consumes large quantities of NAD+ and which is needed for mediating DNA damage repair [62,63,64,65].

In a cohort of 11 patients with brain metastases (originating from breast cancer in four patients), higher hyperpolarized [1-^13^C]lactate z-scores were associated with progression after radiotherapy [66]. However, there were considerable differences in lactate z-scores between metastases originating from different primary tumors. A prediction score based on the lactate z-score reached an AUC for treatment failure prediction of 0.77. It had been shown previously that lactate accumulation, as measured in tumor extracts, could affect radiosensitivity, potentially by affecting a tumor’s antioxidant properties [67].

The first clinical case report in breast cancer used dynamic ^13^C chemical shift imaging (CSI) in a single slice with a temporal resolution of 10 seconds [51], a field-of-view of 160 × 160 mm, a slice thickness of 30 mm, an acquisition matrix of 10 × 10 and reconstruction matrix of 16 × 16. Subsequent studies used IDEAL spiral CSI with a temporal resolution of 4 seconds [33,55]. However, spectral-spatial excitation is being used increasingly as it increases the signal-to-noise ratio for ^13^C-lactate while preserving ^13^C-pyruvate polarization and can be combined with echo-planar or spiral k-space readout trajectories. This pulse sequence was used in the most recent studies on early response assessment in breast cancer and brain metastases [54,66]. For ^13^C-MRI of the breast using both dynamic IDEAL spiral CSI and spectral-spatial excitation, the nominal in-plane resolutions were 5 × 5–6 × 6 mm^2^, and the reconstructed in-plane resolutions were 1.56 × 1.56–1.88 × 1.88 mm^2^ with a slice thickness of 30 mm. The balanced steady-state free precession (bSSFP) sequence has been shown to offer the highest SNR per unit time and can thus improve spatial resolution in dynamic imaging of [1-^13^C]pyruvate and [1-^13^C]lactate [68,69,70,71]. However, bSSFP has yet to be used in a clinical study of breast cancer.

## 7. Concluding Remarks

Over the past two decades, hyperpolarized ^13^C-MRI has shown its potential in oncological imaging in general and in breast cancer imaging in particular. The feasibility of the technique has been demonstrated not only in patients with breast cancer [33,51,54,55] but also with primary tumors of the prostate [20,62], pancreas [72], and kidneys [73,74] and with primary and secondary brain tumors [66,75,76]. A list of clinical trials can be found at https://classic.clinicaltrials.gov/ct2/results?cond=&term=hyperpolarized+carbon&type=&rslt=&age_v=&gndr=&intr=&ttles=&outc=&spons=&lead=&id=&cntry=&state=&city=&dist=&locn=&rsub=&strd_s=&strd_e=&prcd_s=&prcd_e=&sfpd_s=&sfpd_e=&rfpd_s=&rfpd_e=&lupd_s=&lupd_e=&sort= (accessed on 20 June 2023). The focus has been on metabolic imaging with hyperpolarized ^13^C-labeled pyruvate as it is an endogenous molecule that can be polarized to high levels within hours, has a relatively long polarization lifetime in vivo and which produces substantial concentrations of labeled lactate within the lifetime of the hyperpolarization. There is an unmet need for an imaging technique that allows the early identification of those patients undergoing NACT whose tumors are not responding to treatment and are thus unlikely to reach pCR. These patients might benefit from early treatment changes such as changes in drug regimen or earlier surgical resection. The early identification of patients who reach pCR after completion of NACT, on the other hand, could help de-escalation of their treatment. Hyperpolarized ^13^C-MRI has been shown in numerous preclinical and early clinical studies to be a very promising candidate for this purpose. It could easily be combined with ^1^H-MRI, which is currently the most accurate imaging method for local breast cancer staging and response assessment. Early response assessment in patients with breast cancer has been demonstrated using hyperpolarized [1-^13^C]pyruvate after around one week of treatment, and the technique outperformed both DCE MRI with pharmacokinetic modeling and DWI [54]. In addition, metabolic tumor phenotyping might be a second application, as metabolic heterogeneity between tumors of different histopathological and immunohistochemical subtypes has been demonstrated using ^13^C-MRI [33]. However, larger cohorts are needed to validate these initial results, and studies on different tumor subtypes and with different targeted treatments are mandated. There is a need to understand the mechanism(s) by which drugs affect the observed hyperpolarized ^13^C label exchange between pyruvate and lactate, such as the PARP inhibitors, which affect the conversion of pyruvate to lactate by increasing the availability of the coenzyme NADH [54]. An improved understanding of the effects of drugs used in the treatment of patients with breast cancer lactate labeling could help identify ideal scenarios in which to use this imaging technique. For example, resistance to a PI3Kα inhibitor has been shown to be particularly amenable to evaluation using ^13^C-MRI [36], making the imaging technique a likely companion diagnostic tool for breast cancer patients undergoing treatment with this drug. The landscape of drugs being used in the neoadjuvant setting has changed recently with the introduction of the immune checkpoint inhibitor (ICI) pembrolizumab, a monoclonal antibody that blocks the programmed cell death protein 1 (PD-1) receptor, and that has been authorized by the European Medicines Agency for use in patients with locally advanced or early TNBC at high risk of recurrence [77]. Early response assessment is particularly challenging in this case because early immune cell infiltration and pseudo-progression are difficult to distinguish from true progression, especially when restricted to anatomic imaging alone. This can potentially delay the diagnosis of true disease progression and the treatment changes this would entail [78]. Evaluation of different hyperpolarized ^13^C-labeled substrates in patients undergoing immunotherapy has the potential to change the landscape of response assessment, especially in light of conflicting results regarding the ability of [^18^F]FDG-PET to distinguish between progression and pseudo-progression [79,80,81,82]. The first clinical report describing the detection of metastatic prostate cancer responding to ICI treatment using HP ^13^C-MRI has been published [83], and preclinical studies have been performed recently in melanoma and colon carcinoma models [84,85]. Interestingly, one study indicates that hyperpolarized ^13^C-fumarate and its conversion to ^13^C-malate might be a better indicator of response to dual ICI treatment and survival than glycolytic flux imaged using ^13^C-pyruvate [85]. DCE MRI showed an accompanying increase in perfusion/permeability in more treatment-sensitive tumors. Further preclinical and clinical studies are needed to evaluate the ability of different ^13^C-labeled probes to distinguish between true progression and pseudoprogression in breast and other cancers. 

A challenge for the field of hyperpolarized ^13^C-MRI is the technical complexity of the hyperpolarization process, together with the quality checks needed before injection of the short-lived hyperpolarized substrate. These shortcomings need to be addressed together with the relatively high costs that still make hyperpolarized ^13^C-MRI a low throughput imaging technique before ^13^C-MRI becomes more widely applicable in larger clinical studies and trials. The development of PHIP methodology and techniques for centralized production, storage, and transport of hyperpolarized substrates may help in this respect. Uniformity of image acquisition and reconstruction techniques and the quantification of metabolism based on imaging data will also be required in order to obtain large multicentric data sets while maintaining comparability between sites.

## Figures and Tables

**Table 1:** Selected hyperpolarized ^13^C-MRI and ^2^H-MRSI studies in vivo, in vitro, and in the clinic.

Reference	Tumor	Imaging	Results
[25]	Human colorectal (Colo205) and breast adenocarcinoma (MDA-MB-231) xenografts in mice treated with an apoptosis-inducing agent (TRAIL agonist)	^13^C-MRI with hyperpolarized [1-^13^C]pyruvate [^18^F]FDG-PET	At only 24 h after treatment, there was a decrease in lactate labeling, whereas [^18^F]FDG uptake remained unchanged.
[26]	Patient-derived ER+ breast cancer xenografts treated with a PI3Kα inhibitor	^13^C-MRI with hyperpolarized [1-^13^C]pyruvate [^18^F]FDG-PET	^13^C-label exchange between pyruvate and lactate was decreased in drug-sensitive but not in drug-resistant tumors, whereas [^18^F]FDG uptake was unaffected in both.
[32]	TRAMP (Transgenic Adenocarcinoma of Mouse Prostate) mice undergoing radiotherapy	^13^C-MRI with co-polarized [1-^13^C]pyruvate and [^13^C]urea	After 1, 4, and 7 days a decrease in lactate-to-pyruvate conversion was found with a concomitant increase in the hyperpolarized urea signal, indicating increased perfusion. Similar results indicated increased perfusion were obtained with DCE MRI.
[15]	Suspensions of MDA-MB-231 breast cancer cells and mice with subcutaneous MDA-MB-231 tumors following their treatment with doxorubicin	Co-polarized [1-^13^C]pyruvate and [1,4-^13^C_2_]fumarate	Treatment-induced cell death was accompanied by a decrease in ^13^C-label exchange between [1-^13^C]pyruvate and lactate and a concomitant increase in flux between fumarate and malate, which occurred before changes in tumor size.
[33]	Human breast cancer (MDA-MB-231) xenografts	^2^H-MRSI at 7T after injection of [2,3-^2^H_2_]fumarate	At only 48 h after treatment with a TRAIL agonist, the malate-to-fumarate ratio increased significantly.
[34]	One patient with early triple-negative breast cancer undergoing platinum-based neoadjuvant chemotherapy	^13^C-MRI with hyperpolarized [1-^13^C]pyruvate ^1^H-MRI including DCE MRI	In a patient with eventual pathologic complete response (pCR), after the first cycle of neoadjuvant chemotherapy, the lactate-to-pyruvate ratio and the apparent first-order rate constant describing label flux from pyruvate to lactate (*k*_PL_) had decreased while *K*^trans^ on DCE MRI had increased.
[35]	Seven patients with early triple negative or HER2+ breast cancer undergoing neoadjuvant treatment; a subgroup received Olaparib (PARP inhibitor) treatment.	^13^C-MRI with hyperpolarized [1-^13^C]pyruvate ^1^H-MRI including DCE MRI and DWI	At 7–11 days into treatment, an early increase in the lactate-to-pyruvate ratio of ≥20% was observed among patients with pCR, but not those without eventual pCR; neither DCE MRI with pharmacokinetic modeling nor DWI allowed a distinction between these two outcomes.

## Data Availability

Not applicable.
